# What has changed in venous thromboembolism prophylaxis for hospitalized patients over recent decades: review article

**DOI:** 10.1590/1677-5449.002118

**Published:** 2019-01-30

**Authors:** Selma Regina de Oliveira Raymundo, Suzana Margareth Ajeje Lobo, Kassim Mohamede Kassim Hussain, Kassim Guzzon Hussein, Isabela Tobal Secches

**Affiliations:** 1 Faculdade Regional de Medicina de São José do Rio Preto – FAMERP, Hospital de Base, São José do Rio Preto, SP, Brasil.; 2 Hospital Austa, São José do Rio Preto, SP, Brasil.; 3 Faculdade de Medicina em São José do Rio Preto – FACERES, São José do Rio Preto, SP, Brasil.

**Keywords:** venous thromboembolism, prevention & control, anticoagulants

## Abstract

Venous thromboembolism (VTE) is a common disease with high rates of morbidity and mortality and is considered the number one cause of avoidable mortality among hospitalized patients. Although VTE incidence is extremely high in all countries and there is ample evidence that thromboprophylaxis inexpensively reduces the rate of thromboembolic complications in both clinical and surgical patients, a great deal of doubt remains with respect to patient safety with this type of intervention and in relation to the ideal thromboprophylaxis methods. Countless studies and evidence-based recommendations confirm the efficacy of prophylaxis for prevention of VTE and/or patient deaths, but it remains underutilized to this day. This article presents a wide-ranging review of existing prophylaxis methods up to the present, from guidelines and national and international studies of thromboprophylaxis.

## INTRODUCTION

 Deep venous thrombosis (DVT) and pulmonary embolism (PE) are severe public health problems on both the national and international scales, due to the elevated costs associated with acute episodes of venous thromboembolism (VTE) and their long term complications. 1 ^,^ 2 Venous thromboembolism is a serious complication in hospitalized patients and the most common cause of avoidable deaths among these patients, in addition to being the third-ranked cardiovascular cause of death, after coronary disease and strokes. 3

 It is now clear that clinical patients are as much at risk of VTE as surgical patients. Patients who undergo orthopedic surgery or general surgery and those with acute myocardial infarction are at high risk of VTE, and the proportions are almost equal among surgical and clinical patients (22 and 24% respectively). 4 The rate of symptomatic VTE among abdominal surgery patients is in the range of 0.4 to 3.1%. 5

 Immobilization caused by lower limb paralysis or confinement for more than 3 days, severe trauma, and spinal cord trauma can increase the risk of thrombosis by as much as ten times, and the effect is cumulative over time. 6 ^,^ 7 Hospitalized patients with acute clinical diseases are also at significant risk: approximately 10 to 30% of clinical patients develop VTE. 2 Congestive heart failure (CHF) and respiratory failure also increase the risk of thrombosis by up to ten times. 7

 The frequency of thromboembolic complications among hospitalized patients, the adverse consequences of these events, and their economic impact justify prioritizing thromboprophylaxis for the safety of these patients, since it is a significant factor in reducing short and long term morbidity and mortality. However, despite the extremely high incidence of VTE recorded and published in many different studies and the evidence that thromboprophylaxis reduces thromboembolic complications among clinical and surgical patients at low cost, there is still a great deal of doubt with relation to the safety of this type of intervention and to the ideal prophylaxis methods. This situation remains despite more than five decades having passed since the first published study demonstrating that the rate of fatal and symptomatic VTE can be reduced by prophylaxis and almost 30 years since the first evidence-based guideline recommending prophylaxis for the majority of hospitalized patients. 

 This article presents a wide-ranging review of the literature on thromboprophylaxis, based on a systematic analysis of bibliographic references from the PubMed, SciELO, MEDLINE, and LILACS databases for the period 1990-2017 and of selected articles from the CAPES periodicals portal. 

 The objective of this article is to raise awareness of the need for thromboprophylaxis and of strategies for increasing compliance, since, in spite of all efforts, rates are still unsatisfactory all over the world. 

## PROPHYLAXIS METHODS

 There are now many thromboprophylaxis methods available. Non-pharmacological methods, including elastic graduated compression stockings (EGCS), intermittent pneumatic compression (IPC), and venous pumps for feet, have proven effective for reduction of DVT in several patient groups. 2 ^,^ 8 While these mechanical methods do not increase the risk of bleeding, there are few studies of such devices and they appear to be less effective than pharmacological prophylaxis in some groups. 2 According to current evidence, mechanical prophylaxis methods are more often used with patients at high risk of bleeding or in combination with pharmacological prophylaxis, to attempt to increase its efficacy. 2

 Many effective prophylaxis strategies, such as combinations of IPC, EGCS, or pharmacological prophylaxis (acetylsalicylic acid [AAS] or anticoagulants) in high-risk patients, are not included in the guidelines because there are few randomized clinical trials, when compared to any of the options that are recommended, and also because these combinations may not be effective, but are more complex and expensive than simple options. 8 ^-^ 11

 The International Multicenter Trial on PE prevention described by Kakkar is the point of reference for the start of pharmacological prophylaxis and demonstrated that 5,000 international units (IU) of unfractionated heparin (UFH) subcutaneously three times a day reduced the rate of DVT by 25%, while in the group control this reduction was 8%. Fatal PE was eight times less frequent in patients undergoing major surgery. 12 A meta-analysis conducted some years later, of 46 randomized clinical trials including 15,000 surgical patients, demonstrated a reduction in asymptomatic DVT and fatal PE exceeding 60% and significant reductions in their mortality, even when low doses of UFH were used. 13

 Low molecular weight heparin (LMWH) is a more effective prophylaxis option for a large number of patients and tends to be used as a substitute for UFH. 2 ^,^ 14 Low molecular weight heparin has rapid onset of action, requires a single daily dose and, in many countries, is of low cost. Currently, two types of LMWH are available commercially in Brazil: enoxaparin and dalteparin. 

 Oral vitamin K antagonists (VKA), such as warfarin, have been used for prophylaxis in major orthopedic surgery for several decades and, when used correctly, are effective for reducing VTE rates, but they have certain disadvantages: slow onset of action, large variations in dosage from patient to patient, and higher rates of bleeding when prophylaxis is of long duration. 2 ^,^ 8 ^,^ 15

 A synthetic indirect factor Xa inhibitor (fondaparinux) demonstrated greater efficacy than LMWH in more than 7,000 orthopedic surgery patients in a meta-analysis of four randomized studies. 16 It was also effective in studies of prophylaxis for general surgery and clinical patients. 17 ^,^ 18

 For a considerable length of time, the anticoagulants available did not meet criteria for a theoretically ideal anticoagulant, which stimulated researchers to investigate new molecules, with more predictable pharmacokinetics and pharmacodynamics and which would more closely approach the ideal profile of efficacy, safety, and posological comfort. The direct oral anticoagulants 19 rivaroxaban, apixaban, and edoxaban (direct factor X inhibitors) and dabigatran etexilate (a direct thrombin inhibitor) have been approved in some countries for prevention of VTE after total hip arthroplasty (THA) and total knee arthroplasty (TKA). Rivaroxaban was evaluated in four large phase III trials. These double-blind studies, entitled Regulation of Coagulation in Major Orthopedic Surgery Reducing Risk of Deep Venous Thrombosis and Pulmonary Embolism (RECORD) enrolled more than 12,500 elective THA and TKA surgery patients and demonstrated the superiority of rivaroxaban, at a dosage of 10 mg per day, with a reduction of more than 50% in the risk of symptomatic VTE and death, when compared with 40 mg/day of enoxaparin. 20 However, the risk of bleeding with rivaroxaban was 0.7% compared to 0.3% with enoxaparin. 

 The RECORD1 study compared the efficacy of rivaroxaban with enoxaparin during the postoperative period for 5 weeks in THA patients and found a 70% reduction in the relative risk of the primary efficacy outcome when rivaroxaban was used. 21 RECORD2 was a trial designed to study superiority by comparing extended VTE prophylaxis with 10 mg rivaroxaban over 35±4 days with short term prophylaxis with 40 mg of subcutaneous enoxaparin for 10-14 days followed by an oral placebo up to 35±4 days after THA. 22 Extended thromboprophylaxis with rivaroxaban was significantly more effective, with a 79% reduction in the relative risk of the primary efficacy outcome and superior prevention of symptomatic events. 

 RECORD3 was the first study to show a significant reduction in symptomatic VTE among TKA patients, showing the superior efficacy of a 10 mg daily oral dose of rivaroxaban over enoxaparin for 10 to 14 days. 23 In turn, the RECORD4 trial compared the efficacy of 10 mg rivaroxaban per day with 30 mg of enoxaparin, subcutaneously twice a day for a period of 10 to 14 days in patients subjected to TKA, demonstrating that rivaroxaban was not inferior and was associated with a 31% reduction in relative risk of the primary efficacy outcome, in addition to numerical reductions in the secondary efficacy outcomes and symptomatic VTE rates, although these reductions were not significant. 24

 Apixaban is easily absorbed orally and clearance is renal (25-30%) and hepatic (65%). 25 In the ADVANCE1 study, apixaban, at a dosage of 2.5 mg twice a day, was not inferior when compared to enoxaparin (30 mg every 12 hours) in TKA patients, but the outcomes, mortality, and VTE study were similar, while apixaban was associated with a lower rate of bleeding. 26 ^,^ 27

 The ADVANCE2 study, with TKA patients, confirmed that apixaban, at a dosage of 2.5 mg twice a day for 10 to 14 days, was more effective than enoxaparin (40 mg/day), with similar safety. 25 ^,^ 28 The ADVANCE3 study compared apixaban and enoxaparin over 32 to 38 days in THA patients. Apixaban exhibited a statistically superior reduction in the primary outcome (presence of VTE), in death from all causes, and in the composite outcome proximal DVT, non-fatal PE, and VTE-related death. 

 A phase III trial compared edoxaban at a dosage of 30 mg once a day with 20 mg enoxaparin every 12 hours for TKA prophylaxis. The efficacy results for edoxaban were superior to those for enoxaparin at this dosage, and safety (bleeding) was similar. 29

 Dabigatran was used for DVT prophylaxis in TKA and THA surgery in three phase III trials (RE-MODEL, RE-MOBILIZE, and RE-NOVATE), which demonstrated it was not inferior to LMWH. Dabigatran was tested at doses of 150 or 220 mg/day during the postoperative period after THA vs. 40 mg/day enoxaparin (RE-NOVATE) and vs. 30 mg enoxaparin twice a day (RE-MOBILIZE). The primary outcome used for analysis was the rate of DVT, VTE and/or death from all causes. 25 ^,^ 30 ^-^ 32

 It is currently recommended that pharmaceutical prophylaxis should be extended up to 35 days in certain situations, such as orthopedic surgery (TKA, THA, and surgery for hip fractures), because the majority of symptomatic thromboembolic events are diagnosed after discharge and an increased risk of VTE remains for more than 3 months after THA and for more than 1 month after TKA. 33

 A meta-analysis of nine randomized studies with a total of 4,000 major orthopedic surgery patients found a 51% reduction in risk of DVT and 61% for symptomatic VTE with extended prophylaxis, without increased bleeding. 34

 Extended use of fondaparinux for 7 days in patients with hip fractures eliminated asymptomatic DVT and symptomatic VTE. 35 Although prophylaxis lasting from 4 to 6 weeks is superior to prophylaxis only given while in hospital, the optimum duration between 2 and 6 weeks is unknown. Extended prophylaxis is also suggested for patients undergoing major cancer surgery. Two studies showed that among these patients extended prophylaxis lasting 4 weeks was associated with reductions in the DVT rate, when compared with just 1 week. 36

##  PROPHYLAXIS RECOMMENDATIONS FROM THE AMERICAN COLLEGE OF CHEST PHYSICIANS (ACCP) EVIDENCE-BASED CLINICAL PRACTICE GUIDELINES 

 In 1959, publication of a controlled trial of thromboprophylaxis demonstrated that use of an oral anticoagulant in patients undergoing surgical treatment for hip fractures reduced symptomatic VTE and deaths, without increasing clinically significant bleeding. 37 Since that study, hundred of others have been conducted and new prophylaxis options have been evaluated, adopted, and, in some cases, substituted by more effective and safer methods. Since 1986, more than 25 evidence-based guidelines have been published recommending routine thromboprophylaxis for the majority of hospitalized patients. 2 ^,^ 38 Every 4 years, the American College of Chest Physicians (ACCP) publishes guidelines for treatment and prevention of VTE 39 which are a reference worldwide. A quality-based approach has been taken to classification of evidence grades and recommendations since the sixth edition of the ACCP guidelines. 40 The eighth edition (AT8), from 2008, discusses the risks and evidence for thromboprophylaxis for 23 different groups of patients separately, with emphasis on randomized clinical trials 2 (RCT) and classifies recommendations and the methodological quality of the evidence supporting them as follows: 41

Recommendation grades:

- Grade 1: the benefits outweigh harms, burden, and costs;
- Grade 2: individual patient characteristics may lead to different choices.

Evidence level:

- A (high quality): results are from well-planned and conducted RCTs, with parallel groups and adequate controls, appropriate data analysis, and consistent findings;
- B (moderate): from RCTs with small confidence intervals, or cohort, case-control, or observational studies;
- C (low): results from cohort and case-control studies of low quality with a high likelihood of bias.

Recommendation options in favor of or against thromboprophylaxis are described for each group of patients and it is recommended that every hospital should develop its own formal strategy for VTE prevention (Grade 1A) and that thromboprophylaxis should be provided for many hospitalized patients. Use of AAS in isolation was not recommended for any patient group (Grade 1A) and mechanical methods are primarily recommended for patients with a high risk of bleeding (Grade 1A) or as an adjuvant to pharmacological prophylaxis (Grade 2A). 

 In the ninth edition (AT9), from 2012, many recommendations with lower impact substituted the higher impact recommendations in the AT8, because of a more critical evaluation of the inferences underlying evidence and exclusion of specialists with conflicts of interests from the final recommendation process. 42 One limitation of the AT8 was an inconsistent approach to evaluation of risk of bleeding, which was corrected in AT9, where this risk was applied in all chapters. 43 Many new recommendations were added in this edition, but a large number of them had low evidence levels (2C). 

## STUDIES OF PROPHYLAXIS IN CLINICAL PATIENTS

 The Prophylaxis in Medical Patients with Enoxaparin (MEDENOX) trial was the first multicenter randomized study to evaluate efficacy and safety of pharmacological prophylaxis in patients with acute clinical diseases and demonstrate the risk of VTE among these patients. 

 Patients over the age of 40 years admitted with CHF or acute respiratory failure without a need for ventilatory support, or patients with other clinical conditions and at least one risk factor for VTE (age over 75 years, cancer, prior VTE, hormone therapy, obesity, varicose veins, or chronic heart or respiratory failure) were randomized to receive placebo or enoxaparin, daily, at doses of 20 and 40 mg, over periods ranging from 6 to 14 days. The incidence of VTE in the 1,102 patients was significantly lower among those given 40 mg of enoxaparin (5.5%) than in groups given placebo (14.9%) or 20 mg of enoxaparin (15%). The observed benefit of 40 mg of enoxaparin was maintained for 3 months. The incidence of adverse effects did not differ significantly between the placebo group and the enoxaparin group. 44 This study therefore documented the incidence of VTE in clinical patients and also established the efficacy of prophylaxis and the appropriate dose. An increased risk of VTE was also observed after hospital discharge, since the symptomatic DVT rate at 110 days was double the rate observed at 14 days. The objective of the THE-PRINCE 45 randomized study was to determine the efficacy and safety of 40 mg of subcutaneous enoxaparin, once a day, or 5000 IU of UFH three times a day over 10±2 days, in patients with CHF or severe respiratory disease. The incidence of VTE was 8.4% in the enoxaparin group and 10.4% in the UFH group. Enoxaparin was associated with fewer deaths, bleeding events, and adverse effects. The study concluded that enoxaparin was, at least, as effective as UFH for preventing VTE in these clinical patients and offered a better safety profile. 

 In the multicenter, randomized PREVENT 46 study, 1,518 clinical patients were given 5,000 IU of subcutaneous dalteparin once a day and 1,473 were given placebo for 14 days. Dalteparin reduced the rate of VTE to 2.8% without increasing the major hemorrhage rate, compared to the placebo group, with an incidence of 4.9%. This study also showed the need for pharmacological prophylaxis in these patients and established the efficacy and safety of dalteparin. 

 The ARTEMIS study 18 evaluated the efficacy and safety of 2.5 mg fondaparinux used for a period ranging from 6 to 10 days for prevention of VTE in older clinical patients, comparing it with placebo. The incidence of VTE was 10% among patients who received placebo and 5.6% among those given fondaparinux. 

 The PREVAIL study, 47 which assessed use of LMWH in patients with ischemic strokes, observed a DVT prevalence of 20 to 50% and observed that PE was the third most common cause of death. The study assessed 1,762 patients who were unable to walk and had suffered acute ischemic stroke within 48 hours of admission, randomized to receive 5000 IU of UFH twice a day or 40 mg of enoxaparin in a single daily dose, for 10 days. Enoxaparin was more effective than UFH for reduction of VTE in these patients (18% vs. 10%), with the same incidence of major intracranial and extracranial hemorrhages (1%). 

 The International Medical Prevention Registry on Venous Thromboembolism (IMPROVE) was an observational study that evaluated practices for VTE prevention in 15,156 hospitalized clinical patients in twelve countries over 4 years, finding that 50% of the patients received pharmaceutical or mechanical thromboprophylaxis. In the United States, 52% of the patients should have been given thromboprophylaxis, but only 60% of patients with risk factors for VTE actually were given it. In the United States, UFH was the most frequently used drug (20%), whereas LMWH was used most often in the other countries studied (40%). 48

 The EXCLAIM 49 randomized study was conducted with the objective of establishing the appropriate duration of thromboprophylaxis in patients over the age of 40 years hospitalized for acute clinical diseases and immobilized for up to 3 days. In this study, 40 mg enoxaparin was prescribed once a day for 10±4 days, followed by enoxaparin 40 mg once a day or placebo for a further 28±4 days. Compared to placebo, extended treatment with enoxaparin reduced the relative risk of all VTE events by 44% (from 4.9% to 2.8%), of asymptomatic VTE by 34%, and of symptomatic VTE by 73%. In a subset of patients with level 1 immobility (completely confined to bed), VTE was observed in 2.5% of patients given enoxaparin, compared with 4% in the placebo group. The risk of major bleeding was 0.8% in the group given extended enoxaparin and 0.3% in the placebo group. These results demonstrated a 1.5% reduction in the incidence of proximal VTE or PE, at the expense of a 0.5% increase in the incidence of major bleeding. The study concluded that the extended regimen with enoxaparin is safe and effective in clinical patients who remain immobilized. 

 A large, observational, multicenter study assessed the prevalence of VTE in hospitalized clinical patients over the age of 40 years, or over the age of 18 years admitted for surgical treatment or because of traumas, and also studied the proportion of at-risk patients given the appropriate prophylaxis (Epidemiologic International Day for the Evaluation of Patients at Risk of Venous Thrombosis in the Acute Hospital Care Setting, ENDORSE). The study reviewed 37,356 (55%) medical records for clinical patients and 30,827 (45%) for surgical patients against the seventh edition of the ACCP guidelines. The proportion of patients considered at risk of VTE ranged from 36 to 73%. 50 The proportion of at-risk patients who were given appropriate prophylaxis ranged from 2 to 84% in different countries. Among the surgical patients, 64.4% were at risk of VTE, 58.5% were given prophylaxis and, despite 41.5% of the clinical patients being considered at risk, just 39.5% of them were given appropriate prophylaxis. This study demonstrated that, overall, more than 50% of the hospitalized clinical patients needed prophylaxis, but just half of them received it, and that prophylaxis was given to a greater proportion of surgical patients. Furthermore, the study also showed the existence of a large gap in terms of administration of adequate prophylaxis to the population at risk, particularly among clinical patients. 

 After publication of the MEDENOX study, prophylaxis with enoxaparin was once more evaluated in a double-blind, placebo-controlled, randomized study entitled Low-Molecular-Weight Heparin and Mortality in Acutely Ill Medical Patients (LIFENOX), with the objective of evaluating 30-day all-causes mortality after use of 40 mg of subcutaneous enoxaparin for 10±4 days, compared to placebo. 51 The patients enrolled were over the age of 40 years and had been hospitalized due to acute decompensated CHF, active cancer, or severe systemic infection and one additional condition or risk factor (chronic lung disease, obesity, prior history of VTE or age ≥ 60 years). A total of 8,307 patients who spent at least 6 days in hospital were assessed. 

 At 30 days, no significant difference was observed between the enoxaparin and placebo groups in terms of the primary outcome, which was all-causes mortality (4.9% vs. 4.8%, respectively). The study confirmed the need for continued use of pharmacological prophylaxis to prevent VTE and its nonfatal complications in clinically ill hospitalized patients. However, this study suffered from several limitations: 1) the rate of events in the placebo group was lower (4.8%) than expected (7%), and the rate of fatal PE in the placebo group was less than 0.1% at 30 days; 2) patients were younger, with less overweight and had had fewer prior thromboembolic events; and 3) mobility, a major determinant of risk of VTE, was also not assessed, which could have lead to selection of patients with lower rates of events. 

 The MAGELLAN 52 study compared use of 10 mg rivaroxaban per day for extended prophylaxis over 35±4 days with a standard regimen of 10±4 days of enoxaparin (40 mg/day, subcutaneous) in acutely ill clinical patients. A total of 8,101 patients were randomized 1:1 for extended rivaroxaban or enoxaparin. The results of the study confirmed that events related to VTE continue to occur after hospital discharge. There was an increase in the rate of events in the control group from 2.7% at 10 days to 5.7% at 35 days, and a 4.4% rate in the rivaroxaban group, confirming its efficacy for reducing the rate of events; however, the data suggested that the increased risk of major bleeding in this group (1.1% vs. 0.4%) could lead to its use not being recommendable. Clinically relevant bleeding at 35 days was also observed in 4.1% of the patients in the rivaroxaban group and in 1.7% of the control group. 

 The multinational Assessment for VTE management in hospital-Middle East (AVAIL ME) 53 study enrolled clinical and surgical patients with the primary objective of identifying the prevalence of patients at risk of VTE and determining the proportion of hospitalized patients who were given prophylaxis in accordance with the 2004 ACCP guidelines. The authors observed that VTE risk factors and eligibility for prophylaxis were common (exceeding 80%), but rates of prophylaxis and compliance with guidelines were low (37%), and showed that fewer clinical patients were given pharmaceutical prophylaxis than surgical patients. 

 The AVAIL ME Extension Project was published in 2011. Among patients eligible for VTE prophylaxis, 77% were given some type of pharmaceutical prophylaxis, and there was 38% compliance with the AT8 guidelines. 54 In this study, although prophylaxis was apparently being administered with greater frequency than seen in previous reports, a significant percentage of patients were given prophylaxis in the absence of any clear indication of need (78%) or even in the presence of documented contraindications (66%), and clinical patients were given prophylaxis less than surgical patients. 

 An observational cohort study in the United States enrolled 294,896 critically ill patients admitted to intensive care units (ICU) and treated with pharmacological thromboprophylaxis, with mechanical methods, with both, or not given prophylaxis. The main finding of this study is that adult ICU patients on prophylaxis with anticoagulants had a lower risk of mortality than those using mechanical methods or not given any type of prophylaxis. These findings confirm the recommendation of pharmaceutical prophylaxis rather than mechanical prophylaxis for critical patients who do not have any contraindications to anticoagulation. 55

## BRAZILIAN STUDIES OF THROMBOPROPHYLAXIS

 In Brazil, the guidelines for VTE risk factors and risk stratification and thromboembolic recommendations are certified by the Brazilian Medical Association (Associação Médica Brasileira). 56 ^,^ 57

 A study conducted from 1995 to 1999 at the Hospital Naval Marcílio Dias (Rio de Janeiro) analyzed 18,690 patients using Caprini risk stratification, classifying 5% of them as at high risk, 43% as intermediate, and 52% as low risk. The authors observed that the recommended prophylaxis was adopted in 47% of high risk patients, that 33% of moderate risk patients were not given prophylaxis, and that 4.6% of low risk patients were given pharmaceutical prophylaxis, despite not having indications. 58 The 4 years of this pilot study confirmed the viability and value of the register and revealed a considerable increase in use of LMWH, associated with a six fold reduction in the incidence of symptomatic VTE. 

 A new multicenter study was conducted investigating the incidence and distribution of risk factors for VTE in clinical, surgical, and obstetric-gynecological patients in hospital and use of prophylaxis in Brazil. 59 From 1999 to 2001, data were collected on 27,450 patients. The registers showed that approximately one fourth of high-risk patients and half of moderate-risk patients were not given thromboprophylaxis, probably because of a lack of knowledge about risk factors and appropriate prophylactic strategies. No prophylactic measures whatsoever were used for two-thirds of the low-risk patients. 

 Pereira et al. conducted a prospective study with 850 patients admitted to the Hospital de Roraima to investigate whether DVT prophylaxis was being used correctly, according to the Caprini model. 60 Overall, 67% of the sample were clinical patients and 58% were classified as at medium or high risk of developing DVT. Just 24% of the patients who needed thromboprophylaxis were given it and the thromboprophylaxis provided was only classified as adequate in 20%. The authors concluded that ongoing education programs were needed. 

 Rocha et al. 61 ^,^ 62 embarked on a project to implement a VTE prophylaxis program for clinical patients, setting up a commission, holding lectures, and distributing algorithms based on the Brazilian guidelines and assessing its impact on adequacy of thromboprophylaxis in hospitals in Salvador, in three phases. In the first phase, conducted in 2005, use and adequacy of prophylaxis were assessed with hospitalized clinical patients. In the second, 12-month phase, starting in 2007, a prophylaxis program was implemented. In the third phase, in 2008, the program’s impact was evaluated. The authors concluded that risk factors are frequent among clinical patients, that there is great variation in the prophylaxis prescribed at public and private hospitals, and that only a minority of hospitalized clinical patients who were candidates for prophylaxis were given it at the correct dosage. They also concluded that ongoing education lectures and passive distribution of VTE prophylaxis algorithms were ineffective for improving utilization of prophylaxis, but did improve its appropriateness. 61 ^,^ 62

 Okuhara et al. 63 conducted a study with 296 patients in hospital for vascular and orthopedic surgical procedures, to determine the incidence of DVT and the quality of the prophylaxis provided. The overall incidence of DVT was 7.5%. When put into risk groups, 15% were classified as moderate risk, 24% as high risk and 50% as very high risk. Prophylaxis was correct in just 58%. The rates of appropriate prophylaxis were 72% for both the high and the very high risk groups. Excessive use of pharmacological prophylaxis was observed in 69% and 61% of the low and moderate risk groups, respectively. Although the majority of patients were considered high and very high risk, prophylaxis use continues to be deficient in medical practice. 63

 Finally, the most recent cross-sectional study involving analysis of medical records in the city of Curitiba (published in 2017) compared use or not of prophylaxis within surgical and clinical specialties according to their VTE risk factors and showed that just 66% of the patients were given prophylaxis. In this study, 93% of clinical patients were given prophylaxis compared with 44% in the surgical group. Clinical patients at moderate and high risk were given more prophylaxis than surgical patients. 64

##  STRATEGIES TO INCREASE COMPLIANCE WITH GUIDELINES AND APPRORIATE USE OF THROMBOPROPHYLAXIS 

 Several different studies have reported evidence that alerting health professionals to patients at risk of VTE increases the probability that prophylaxis will be used. 

 There are many different approaches and strategies. Simple didactic education and passive distribution of guidelines based on evidence alone are ineffective. 65 ^,^ 66 Multiple and repeated application of different techniques to teach content, systems for alerting professionals with reminders to conduct risk assessments, and audits are all needed and it appears that a combination of these systems is in fact the most effective approach. One technique for increasing efficacy and use of thromboprophylaxis is implementation of electronic alert systems, which have been in use for 17 years. A randomized controlled study published in 2001, investigating a computerized system that automatically reminded physicians of the need for prophylaxis in hospitalized patients, showed that prophylactic heparin was administered to 32.2% of patients by a group of professionals using the electronic system and to 18.9% of patients by a group that was not using it. 67

 In the United Kingdom, a computerized Clinical Decision Support (CDS) system was used to instruct and guide professionals to prescribe prophylaxis appropriately. The increases in compliance in response to reminders on computers are still modest, 68 but electronic alerts and computerized CDS systems do increase rates of prescription to hospitalized clinical patients. 69 ^,^ 70

 A cross-sectional study conducted in two phases (before and after implementation of a new VTE prophylaxis protocol) was conducted in a hospital in Porto Alegre (Brazil) to evaluate the impact on prophylaxis of a computerized CDS system combined with instructional seminars. Adequacy of prophylaxis increased from 46.2% to 57.9% in the before and after comparison between the two periods, and the increases were greatest among patients with cancer (18.1% to 44.1%) and those with three or more risk factors (25% to 42.9%). 71

 Despite the many initiatives and the increased use of VTE risk assessment, prophylaxis is still being underutilized today and there is also evidence of low compliance with published guidelines. 72 ^,^ 73

 Lau & Haut conducted a MEDLINE search to identify studies published from 2001 to 2012 that evaluated the different types of interventions designed to improve use of VTE prophylaxis in hospitalized patients, classified according to the following parameters: exclusively educational, paper-based, computerized, real time auditing, or a combination of interventions. 74

 There is robust evidence from many different high quality studies demonstrating the effectiveness of VTE prophylaxis in specific populations; however, risk stratification is needed to ensure that prophylaxis is targeted to the appropriate patients and, even then, prophylaxis rates remain sub-optimal and VTE continues to be a problem for patient safety. Furthermore, there is a lack of evidence to show which specific interventions are effective for increasing these prophylaxis rates. Education of professionals with no other interventions is not the best mechanism for increasing prophylaxis utilization. 

 Although derived from non-randomized studies without control patients and, therefore, considered of low quality, there is evidence to suggest that education combined with other strategies for quality improvement and technological initiatives, such as reminders and obligatory computerized CDS systems, is probably the best strategy to promote the practice of prophylaxis use, thereby avoiding harm to patients caused by VTE. 
