# Supplementary material for: What has changed in venous thromboembolism prophylaxis for hospitalized patients over recent decades: review article
Source: J Vasc Bras. 2019 Jan 30;18:e20180021. doi: 10.1590/1677-5449.002118 (PMC6542320; doi:10.1590/1677-5449.002118)
Supplement: Translation

# Translation: O que mudou nas últimas décadas na profilaxia do tromboembolismo venoso em pacientes internados: artigo de revisão

**Authors:** Selma Regina de Oliveira Raymundo, Suzana Margareth Ajeje Lobo, Kassim Mohamede Kassim Hussain, Kassim Guzzon Hussein, Isabela Tobal Secches

**Affiliations:** 1 Faculdade Regional de Medicina de São José do Rio Preto – FAMERP, Hospital de Base, São José do Rio Preto, SP, Brasil.; 2 Hospital Austa, São José do Rio Preto, SP, Brasil.; 3 Faculdade de Medicina em São José do Rio Preto – FACERES, São José do Rio Preto, SP, Brasil.

**Keywords:** tromboembolismo venoso, prevenção & controle, anticoagulantes

## Abstract

O tromboembolismo venoso (TEV) é uma doença frequente e de alta morbimortalidade, sendo considerada a maior causa evitável de mortalidade em pacientes hospitalizados. Apesar da incidência altíssima de TEV em todos os países e das evidências de que a tromboprofilaxia reduz as complicações tromboembólicas em pacientes clínicos e cirúrgicos, e a custo baixo, persistem grandes dúvidas quanto à segurança desse tipo de intervenção nos pacientes e quanto à tromboprofilaxia ideal. Inúmeros estudos e recomendações baseadas em evidências comprovam a eficácia da profilaxia na prevenção do TEV e/ou da morte dos pacientes, mas ainda hoje ela é subutilizada. Neste artigo, apresentamos uma ampla revisão dos métodos de profilaxia existentes até os dias atuais, publicados em diretrizes e estudos nacionais e internacionais sobre tromboprofilaxia.

## INTRODUÇÃO

 A trombose venosa profunda (TVP) e a embolia pulmonar (EP) constituem graves problemas de saúde pública nacional e internacional, devido aos altos custos associados a episódios agudos de tromboembolismo venoso (TEV) e a complicações a longo prazo 1 ^,^ 2 . O TEV é uma séria complicação em pacientes hospitalizados e a causa mais frequente de morte evitável nesses pacientes, além de ser a terceira causa de morte de origem cardiovascular, após doença coronariana e acidente cerebrovascular 3 . 

 É evidente nos dias de hoje que pacientes clínicos são de risco para TEV tanto quanto pacientes cirúrgicos. Pacientes submetidos a cirurgia ortopédica, a cirurgia geral e com infarto agudo do miocárdio apresentam alto risco de TEV, e a proporção é quase igual nos pacientes cirúrgicos e clínicos (22% e 24% respectivamente) 4 . Entre pacientes submetidos a cirurgias abdominais, TEV sintomático tem uma taxa de 0,4 a 3,1% 5 . 

 A imobilização devido à paralisia de membros inferiores ou repouso por mais de 3 dias, assim como trauma grave e trauma raquimedular, aumenta o risco de trombose em até dez vezes, com efeito cumulativo com o tempo 6 ^,^ 7 . Pacientes hospitalizados com doenças clínicas agudas também apresentam risco significativo: aproximadamente 10 a 30% dos pacientes clínicos podem evoluir com TEV 2 . A insuficiência cardíaca congestiva (ICC) e insuficiência respiratória também aumentam o risco trombótico em até dez vezes 7 . 

 A frequência de complicações tromboembólicas em pacientes hospitalizados, as consequências adversas desses eventos e seu impacto econômico justificam a prioridade da tromboprofilaxia para segurança desses pacientes e constitui fator significativo para reduzir a morbidade a curto e longo prazo e a mortalidade. Entretanto, apesar da incidência altíssima de TEV registrada e publicada em vários estudos e das evidências de que a tromboprofilaxia reduz complicações tromboembólicas em pacientes clínicos e cirúrgicos a custo baixo, persistem grandes dúvidas quanto à segurança desse tipo de intervenção e quanto à forma ideal de profilaxia. Isso ocorre mesmo após mais de cinco décadas desde o primeiro estudo publicado demonstrando que o TEV sintomático e fatal poderia ser reduzido com a profilaxia, e quase 30 anos após a primeira diretriz baseada em evidências recomendando profilaxia na maioria dos pacientes hospitalizados. 

 Neste artigo, os autores fazem ampla revisão da literatura sobre tromboprofilaxia, com análise sistemática das referências bibliográficas nas bases de dados PubMed, SciELO, MEDLINE e LILACS no período de 1990-2017 e de artigos selecionados dos periódicos CAPES. 

 O objetivo deste artigo consiste em alertar sobre a necessidade da tromboprofilaxia e as estratégias para aumentar sua adesão pois, apesar de todos os esforços, esta ainda permanece insatisfatória no mundo todo. 

## MÉTODOS DE PROFILAXIA

 Muitas opções de tromboprofilaxia estão atualmente disponíveis. Métodos não farmacológicos, incluindo meias elásticas de compressão graduada (MECG), compressão pneumática intermitente (CPI) e bombas venosas para pés, têm-se mostrado eficientes na redução de TVP em vários grupos de pacientes 2 ^,^ 8 . Embora esses métodos mecânicos não aumentem o risco de sangramento, existem poucos estudos sobre esses dispositivos, e eles parecem ser menos eficazes que a profilaxia farmacológica em alguns grupos 2 . Segundo evidências atuais, opções de profilaxia mecânica são mais utilizadas para pacientes com alto risco de sangramento ou combinadas com profilaxia farmacológica para tentar aumentar sua efetividade 2 . 

 Várias estratégias de profilaxia efetivas, como a combinação de CPI, MECG ou farmacoprofilaxia [ácido acetilsalicílico (AAS) ou anticoagulantes] em pacientes de alto risco, não estão incluídas nas diretrizes, porque existem poucos ensaios clínicos randomizados, quando comparados a qualquer das opções recomendadas, e também porque essas combinações podem não ser mais efetivas, além de serem mais complexas e caras que as opções simples 8 ^-^ 11 . 

 O *International Multicenter Trial*, conduzido por Kakkar, foi o ponto de referência para o início da farmacoprofilaxia, demonstrando que heparina não fracionada (HNF) 5.000 unidades internacionais (UI) subcutânea três vezes ao dia reduziu a TVP em 25%, enquanto que no grupo controle essa redução foi de 8%. A EP fatal diminuiu oito vezes em pacientes submetidos a cirurgias maiores 12 . Uma metanálise realizada alguns anos após, com 46 ensaios clínicos randomizados, incluindo 15.000 pacientes cirúrgicos, demonstrou uma redução maior que 60% em TVP assintomática e EP fatal, bem como uma diminuição significante na mortalidade mesmo quando utilizadas baixas doses de HNF 13 . 

 A heparina de baixo peso molecular (HBPM) é uma opção mais eficiente de profilaxia para um grande número de pacientes, tendendo a substituir a HNF 2 ^,^ 14 . A HBPM tem rápido início de ação, dose única diária e, em muitos países, custo baixo. No Brasil, atualmente, são comercializados dois tipos de HBPM: enoxaparina e dalteparina. 

 Os antagonistas da vitamina K (AVK) orais, como a varfarina, têm sido usados na profilaxia de cirurgias ortopédicas maiores por várias décadas e, quando utilizados de forma correta, são eficazes na redução do TEV, porém com algumas desvantagens: início de ação prolongada, variação grande na dose entre os pacientes e taxas maiores de sangramento na profilaxia estendida 2 ^,^ 8 ^,^ 15 . 

 O inibidor indireto sintético do fator Xa (fondaparinux) mostrou maior eficácia que a HBPM em mais de 7.000 pacientes submetidos a cirurgia ortopédica em uma metanálise que incluiu quatro estudos randomizados 16 . Também foi eficaz em estudos de profilaxia em cirurgia geral e em pacientes clínicos 17 ^,^ 18 . 

 Por muito tempo, os anticoagulantes disponíveis não preencheram os critérios de um anticoagulante teoricamente ideal, o que motivou pesquisas de novas moléculas, com farmacocinética e farmacodinâmica mais previsível e que se aproximassem desse perfil de eficácia, segurança e conforto posológico. Entre os anticoagulantes orais diretos 19 , rivaroxabana, apixabana e edoxabana (inibidores diretos do fator X) e etexilato de dabigatrana (inibidor direto da trombina) estão aprovados em alguns países para prevenção de TEV após artroplastia total de quadril (ATQ) e de joelho (ATJ). A rivaroxabana foi avaliada em quatro grandes estudos fase III. Esses estudos duplo-cegos, denominados *Regulation of Coagulation in Major Orthopaedic surgery Reducing Risk of Deep Venous Thrombosis and Pulmonary Embolism* (RECORD), incluíram mais de 12.500 pacientes submetidos a cirurgia eletiva de ATQ e ATJ e demonstraram a superioridade da rivaroxabana, na dose de 10 mg diária, com redução superior a 50% de risco nas taxas de TEV sintomática e de morte, quando comparada à enoxaparina 40 mg/dia 20 . Contudo, o risco de sangramento foi de 0,7% com rivaroxabana contra 0,3% com enoxaparina. 

 O estudo RECORD1 comparou a eficácia da rivaroxabana com a da enoxaparina no pós-operatório durante 5 semanas em pacientes submetidos a ATQ e evidenciou redução de 70% do risco relativo no desfecho primário de eficácia com rivaroxabana 21 . O RECORD2 foi um estudo de superioridade para comparar a profilaxia estendida do TEV com 10 mg de rivaroxabana durante 35±4 dias com a profilaxia de curto prazo utilizando 40 mg de enoxaparina subcutânea durante 10-14 dias seguida por placebo oral até 35±4 dias após ATQ 22 . A tromboprofilaxia estendida com rivaroxabana foi significativamente mais eficaz, com redução de 79% do risco relativo no desfecho primário de eficácia e na prevenção de eventos sintomáticos. 

 O RECORD3 foi o primeiro estudo que mostrou a diminuição significativa do TEV sintomático em pacientes submetidos a ATJ, evidenciando a eficácia superior de uma posologia oral diária de 10 mg de rivaroxabana comparada à enoxaparina durante 10 a 14 dias 23 . O RECORD4, por sua vez, comparou a eficácia de 10 mg de rivaroxabana ao dia com a de 30 mg de enoxaparina subcutânea duas vezes ao dia num período de 10 a 14 dias em pacientes submetidos a ATJ, demonstrando a não inferioridade da rivaroxabana e uma redução de 31% do risco relativo no desfecho de eficácia primária, além de reduções numéricas nos desfechos de eficácia secundária e no TEV sintomático, embora essas reduções não tenham sido significativas 24 . 

 A apixabana tem fácil absorção oral e apresenta eliminação renal (25-30%) e hepática (65%) 25 . No estudo ADVANCE1, a apixabana, na dose de 2,5 mg duas vezes ao dia, não se mostrou inferior quando comparada à enoxaparina (30 mg de 12 em 12 horas) em pacientes submetidos a ATJ, porém os desfechos e as taxas de mortalidade e TEV foram semelhantes, com menor taxa de sangramento com apixabana 26 ^,^ 27 . 

 O estudo ADVANCE2, com pacientes submetidos a ATJ, comprovou que a apixabana, na dose de 2,5 mg duas vezes ao dia por 10 a 14 dias, foi mais eficaz que a enoxaparina (40 mg/dia), com segurança semelhante 25 ^,^ 28 , e o estudo ADVANCE3 comparou a apixabana e enoxaparina durante 32 a 38 dias em pacientes submetidos a ATQ. A apixabana mostrou uma redução estatisticamente superior no desfecho primário (presença de TEV), na morte por todas as causas e no conjunto da TVP proximal, EP não fatal e morte relacionada com TEV. 

 A edoxabana na dose de 30 mg uma vez ao dia foi comparada à enoxaparina 20 mg de 12 em 12 horas em um estudo fase III de profilaxia em ATJ. Os resultados de eficácia da edoxabana foram superiores aos da enoxaparina nessa dosagem, e os de segurança (sangramentos) foram similares 29 . 

 A dabigatrana foi usada para profilaxia de TVP em ATJ e ATQ em três estudos fase III (RE-MODEL, RE-MOBILIZE e RE-NOVATE), que demonstraram a não inferioridade em comparação à HBPM. A dabigatrana foi testada nas doses de 150 ou 220 mg/dia no pós-operatório de ATQ *versus* enoxaparina 40 mg/dia (RE-NOVATE) e *versus* enoxaparina 30 mg duas vezes ao dia (RE-MOBILIZE). A análise do desfecho primário foi a taxa de TVP, TEV e/ou morte por todas as causas 25 ^,^ 30 ^-^ 32 . 

 Atualmente, sugere-se a extensão da profilaxia medicamentosa por até 35 dias em algumas situações, como cirurgias ortopédicas (ATJ, ATQ e cirurgia para fratura de quadril), pois a maioria dos eventos tromboembólicos sintomáticos é diagnosticada após a alta e o risco aumentado de TEV continua por mais de 3 meses após ATQ e por mais de 1 mês após ATJ 33 . 

 Uma metanálise com nove estudos randomizados, abrangendo 4.000 pacientes submetidos a cirurgia ortopédica maior, evidenciou redução de risco de 51% para TVP e de 61% para TEV sintomático com profilaxia estendida, sem aumento no sangramento 34 . 

 O uso estendido de fondaparinux por 7 dias em pacientes com fratura de quadril eliminou a TVP assintomática e o TEV sintomático 35 . Embora a profilaxia de 4 a 6 semanas de profilaxia seja superior à profilaxia usada apenas no hospital, a duração ótima entre 2 e 6 semanas é incerta. Em pacientes submetidos a cirurgia oncológica de grande porte, a profilaxia estendida também é sugerida. Em dois estudos, a profilaxia estendida por 4 semanas nesses pacientes mostrou redução na taxa de TVP quando comparada a 1 semana apenas 36 . 

##  DIRETRIZES DE PROFILAXIA DAS *AMERICAN COLLEGE OF CHEST PHYSICIANS (ACCP) EVIDENCE-BASED CLINICAL PRACTICE GUIDELINES*

 Em 1959, a publicação de ensaio controlado de tromboprofilaxia demonstrou que o uso de anticoagulante oral em pacientes submetidos a tratamento cirúrgico de fraturas de quadril reduziu TEV sintomático e morte, sem aumento de sangramento clinicamente significativo 37 . A partir desse estudo, centenas de outros foram realizados e novas opções de profilaxia têm sido avaliadas, usadas, e, em alguns casos, substituídas por métodos mais efetivos e seguros. Desde 1986, foram publicadas mais de 25 diretrizes baseadas em evidência recomendando tromboprofilaxia de rotina na maioria dos pacientes hospitalizados 2 ^,^ 38 . A cada 4 anos, o *American College of Chest Physicians* (ACCP) tem publicado diretrizes para tratamento e prevenção do TEV 39 que são referência no mundo todo. A partir da sexta edição das diretrizes do ACCP 40 , adotou-se uma abordagem de qualidade na classificação de evidências e graus de recomendações. A oitava edição (AT8), de 2008, discute, separadamente, os riscos para 23 grupos de pacientes e as evidências para tromboprofilaxia com ênfase em ensaios clínicos randomizados 2 (ECR), e classifica as recomendações e a qualidade metodológica da evidência em 41 : 

Grau de recomendação:

- Grau 1: os benefícios superam os riscos, encargos e custos;
- Grau 2: características individuais do paciente podem levar a diferentes escolhas.

Nível de evidência:

- A (alta qualidade): resultados são provenientes de ECR bem planejados e conduzidos, com grupos paralelos e controles adequados, análise de dados adequada e achados consistentes;
- B (moderada): provenientes de ECR com intervalo de confiança pequeno, estudos de coorte, caso-controle ou observacionais;
- C (baixa): resultado de estudos de coortes e caso-controle com baixa qualidade e alta probabilidade de viés.

Opções de recomendações a favor ou contra tromboprofilaxia foram descritas para cada grupo de pacientes, sendo recomendado que cada hospital desenvolvesse uma estratégia formal própria para prevenção de TEV (Grau 1A) e que a tromboprofilaxia deveria ser realizada em muitos pacientes hospitalizados. Não foi recomendado uso de AAS isolado para qualquer grupo de paciente (Grau 1A), e recomendou-se o uso de métodos mecânicos, principalmente para pacientes com alto risco de sangramento (Grau 1A) ou como adjuvante à farmacoprofilaxia (Grau 2A). 

 Na nona edição (AT9), de 2012, muitas recomendações de impacto menor substituíram as de maior impacto da AT8, devido à avaliação mais crítica de inferências que resultam em evidências e exclusão de especialistas com conflito de interesses no processo final das recomendações 42 . Uma limitação da AT8 foram as abordagens inconsistentes para avaliar o risco de sangramento, o que foi corrigido na AT9, sendo esse risco aplicado em todos os capítulos 43 . Nessa edição, muitas novas recomendações foram incluídas, mas várias delas apresentavam baixo nível de evidência (2C). 

## ESTUDOS SOBRE PROFILAXIA EM PACIENTES CLÍNICOS

 O *Prophylaxis in Medical Patients with Enoxaparin* (MEDENOX) foi o primeiro estudo multicêntrico randomizado que avaliou a eficácia e a segurança da farmacoprofilaxia em pacientes com doença clínica aguda e demonstrou o risco de TEV nesses pacientes. 

 Pacientes com mais de 40 anos admitidos com ICC, insuficiência respiratória aguda sem necessidade de suporte ventilatório, ou pacientes com outras condições clínicas e ao menos um fator de risco para TEV (idade superior a 75 anos, câncer, TEV prévio, hormonioterapia, obesidade, veias varicosas, insuficiência cardíaca ou respiratória crônica) foram randomizados para receberem placebo ou enoxaparina, diariamente, nas doses de 20 e 40 mg, durante um período de 6 a 14 dias. A incidência de TEV nos 1.102 pacientes foi significativamente menor no grupo que recebeu 40 mg de enoxaparina (5,5%) do que nos grupos que receberam placebo (14,9%) e enoxaparina 20 mg (15%). O benefício observado com 40 mg de enoxaparina foi mantido por 3 meses. A incidência de efeitos adversos não diferiu significativamente entre o grupo placebo e o grupo enoxaparina 44 . Esse estudo documentou, portanto, a incidência de TEV em pacientes clínicos e também estabeleceu a eficácia e a dose apropriada da profilaxia. Um risco aumentado de TEV após alta hospitalar também foi observado, visto que a taxa de TVP sintomática em 110 dias dobrou em relação à observada em 14 dias. O objetivo do estudo randomizado THE-PRINCE 45 foi determinar a eficácia e a segurança da enoxaparina 40 mg subcutânea, uma vez ao dia, ou de 5000 UI de HNF três vezes ao dia durante 10±2 dias, em pacientes com ICC ou doença respiratória severa. A incidência de TEV foi de 8,4% no grupo enoxaparina e de 10,4% no grupo HNF. Com enoxaparina constataram-se menos mortes e sangramentos e menos efeitos adversos. O estudo concluiu que enoxaparina é, no mínimo, tão efetiva quanto a HNF para prevenir TEV nesses pacientes clínicos e apresenta um melhor perfil de segurança. 

 No estudo multicêntrico e randomizado PREVENT 46 , 1.518 pacientes clínicos receberam dalteparina 5.000 UI subcutânea uma vez ao dia e 1.473 receberam placebo por 14 dias. Dalteparina reduziu a taxa de TEV para 2,8%, sem aumento de hemorragia maior, comparado ao grupo placebo, com incidência de 4,9%. Esse estudo também mostrou a necessidade da farmacoprofilaxia nesses pacientes e estabeleceu a eficácia e a segurança da dalteparina. 

 O estudo ARTEMIS 18 avaliou a eficácia e a segurança do fondaparinux 2,5 mg, quando utilizado por um período de 6 a 10 dias, na prevenção de TEV em pacientes clínicos mais idosos,, em comparação ao placebo. A incidência de TEV foi de 10% nos pacientes que receberam placebo e de 5,6% nos que receberam fondaparinux. 

 O estudo PREVAIL 47 , que avaliou o uso de HBPM em pacientes com acidente vascular cerebral isquêmico (AVCi), encontrou uma prevalência de TVP de 20 a 50% e observou que a EP foi a terceira causa de morte. Foram avaliados 1.762 pacientes que não andavam e apresentavam AVCi agudo com até 48 horas da admissão, os quais foram randomizados para receber HNF 5000 UI duas vezes ao dia ou enoxaparina 40 mg, em dose única diária, por 10 dias. A enoxaparina foi mais efetiva na redução de TEV nesses pacientes, de 18% contra 10% da HNF, com a mesma incidência de hemorragia intracraniana e extracraniana maior (1%). 

 O *International Medical Prevention Registry on Venous Thromboembolism* (IMPROVE), um estudo observacional que avaliou práticas de prevenção de TEV em 15.156 pacientes clínicos hospitalizados em doze países por 4 anos, verificou que 50% dos pacientes receberam tromboprofilaxia medicamentosa ou mecânica. Nos EUA, 52% dos pacientes deveriam ter recebido tromboprofilaxia, mas somente 60% dos pacientes com fatores de risco para TEV a receberam. A HNF foi a mais usada nos EUA (20%), enquanto HBPM foi mais utilizada em outros países (40%) 48 . 

 O estudo randomizado EXCLAIM 49 foi realizado com o objetivo de estabelecer a duração apropriada da tromboprofilaxia em pacientes com mais de 40 anos hospitalizados com doença clínica aguda e imobilização de até 3 dias. Nesse estudo, Foi prescrita enoxaparina 40 mg uma vez ao dia por 10±4 dias, seguida de enoxaparina 40 mg uma vez ao dia ou placebo por mais 28±4 dias. Comparado ao placebo, a terapia estendida com enoxaparina reduziu o risco relativo de todos os eventos de TEV em 44% (de 4,9% para 2,8%), de TEV assintomático em 34%, e de TEV sintomático em 73%. No subgrupo de pacientes com imobilidade nível 1 (repouso no leito completo), TEV foi observado em 2,5% dos pacientes com enoxaparina comparado com 4% no grupo placebo. O risco de sangramento maior foi de 0,8% no grupo com enoxaparina estendida e de 0,3% no grupo placebo. Esses resultados demonstraram um decréscimo de 1,5% na incidência de TEV proximal ou de EP às custas de um aumento de 0,5% na incidência de sangramento maior. O estudo concluiu que o regime estendido com enoxaparina é eficaz e seguro em pacientes clínicos que permanecem imobilizados. 

 Um grande estudo multicêntrico observacional avaliou a prevalência de TEV em pacientes hospitalizados clínicos com mais de 40 anos, ou maiores de 18 anos admitidos para tratamento cirúrgico ou devido a traumas, e a proporção de pacientes de risco que recebiam profilaxia apropriada (*Epidemiologic International Day for the Evaluation of Patients at Risk of Venous Thrombosis in the Acute Hospital Care Setting,* ENDORSE). Foram revisados 37.356 (55%) prontuários de pacientes clínicos e 30.827 (45%) de pacientes cirúrgicos, e foi usada a sétima edição das diretrizes do ACCP. Os pacientes considerados de risco para TEV variavam entre 36 e 73% 50 . A proporção de pacientes de risco que receberam profilaxia apropriada variou de 2 a 84% em diferentes países. Entre pacientes cirúrgicos, 64,4% eram de risco para TEV, 58,5% receberam profilaxia e, apesar de 41,5% dos pacientes clínicos serem considerados de risco, somente 39,5% deles receberam profilaxia apropriada. Esse estudo demonstrou que, no total, mais de 50% dos pacientes clínicos hospitalizados necessitam de profilaxia mas somente metade deles a recebe, e que a profilaxia é realizada em maior extensão nos pacientes cirúrgicos. Além disso, o estudo mostrou a existência de uma grande lacuna na administração adequada de profilaxia na população de risco, principalmente em pacientes clínicos. 

 Desde a publicação do estudo MEDENOX, a profilaxia com enoxaparina foi novamente avaliada em um estudo randomizado, duplo-cego, placebo controlado denominado *Low-Molecular-Weight Heparin and Mortality in Acutely Ill Medical Patients* (LIFENOX), com o objetivo de verificar a mortalidade em 30 dias devido a qualquer causa após o uso de enoxaparina 40 mg subcutânea durante 10±4 dias, comparada ao placebo 51 . Foram incluídos pacientes acima de 40 anos de idade hospitalizados devido a ICC descompensada aguda, neoplasia ativa ou infecção sistêmica severa e com uma condição ou fator de risco adicional (doença pulmonar crônica, obesidade, história prévia de TEV ou idade ≥ 60 anos). Foram avaliados 8.307 pacientes internados por pelo menos 6 dias. 

 Em 30 dias, nenhuma diferença significativa foi observada entre os grupos enoxaparina e placebo no resultado primário de mortalidade por todas as causas (4,9% *versus* 4,8%, respectivamente). O estudo confirmou a necessidade do uso continuado de farmacoprofilaxia para prevenir TEV e suas complicações não fatais em pacientes hospitalizados clinicamente doentes. Todavia, esse estudo apresentou várias limitações: 1) a taxa de eventos no grupo placebo foi menor (4,8%) que a esperada (7%), e a taxa de EP fatal no grupo placebo foi menor que 0,1% em 30 dias; 2) os pacientes eram mais jovens, com menos sobrepeso e tinham menos eventos tromboembólicos prévios; 3) a mobilidade, um risco determinante maior para TEV, também não foi avaliada, o que pode ter levado à seleção de pacientes com taxas de eventos menores. 

 O estudo MAGELLAN 52 comparou o uso de rivaroxabana 10 mg ao dia de profilaxia estendida por 35±4 dias com um regime padrão de 10±4 dias de enoxaparina (40 mg/dia, subcutânea) em pacientes clínicos agudamente doentes. Um total de 8.101 pacientes foi randomizado 1:1 para rivaroxabana estendida ou enoxaparina. Os resultados desse estudo confirmaram que eventos relacionados ao TEV continuaram a ocorrer após alta hospitalar. Houve um aumento de eventos no grupo controle de 2,7% em 10 dias para 5,7% em 35 dias e taxa de 4,4% no grupo rivaroxabana, comprovando sua eficácia em diminuir eventos; entretanto, dados sugerem que o risco aumentado de sangramento maior nesse grupo (1,1% *versus* 0,4%) pode não sugerir seu uso. Sangramento clinicamente relevante em 35 dias também foi observado em 4,1% dos pacientes no grupo rivaroxabana e em 1,7% no grupo controle. 

 O estudo multinacional *Assessment for VTE management in hospital Middle East* (AVAIL ME) 53 foi realizado em pacientes clínicos e cirúrgicos com o objetivo primário de identificar a prevalência de pacientes com risco de TEV e definir a taxa de pacientes hospitalizados que receberam profilaxia de acordo com diretrizes de 2004 do ACCP. Os autores observaram que os fatores de risco e elegibilidade para profilaxia de TEV são comuns (mais que 80%), mas a profilaxia e aplicação das diretrizes foi baixa (37%), e mostraram que pacientes clínicos receberam menos profilaxia medicamentosa do que pacientes cirúrgicos. 

 O AVAIL ME Extension Project foi publicado em 2011. Dos pacientes elegíveis para receber profilaxia de TEV, 77% receberam alguma profilaxia medicamentosa, com 38% de adesão às diretrizes da AT8 54 . Nesse estudo, embora aparentemente a profilaxia tivesse sido administrada mais frequentemente que em relatos anteriores, em uma porcentagem significativa de pacientes foi realizada profilaxia sem qualquer indicação clara (78%) ou na presença de contraindicações documentadas (66%), e os pacientes clínicos receberam menos profilaxia que os cirúrgicos. 

 Um estudo de coorte observacional nos EUA incluiu 294.896 pacientes criticamente doentes internados em unidade de terapia intensiva (UTI) e tratados com tromboprofilaxia farmacológica, com métodos mecânicos, com ambos, ou que não receberam profilaxia. O principal achado desse estudo é que pacientes adultos em UTI em uso de profilaxia com anticoagulantes apresentavam menor risco de mortalidade do que os que usavam métodos mecânicos ou que não utilizaram profilaxia alguma. Esses achados confirmam a recomendação da profilaxia medicamentosa em vez de profilaxia mecânica para pacientes críticos que não possuem contraindicação à anticoagulação 55 . 

## ESTUDOS BRASILEIROS DE TROMBOPROFILAXIA

 No Brasil, as diretrizes que abordam os fatores e a estratificação de risco de TEV e as recomendações tromboembólicas são apoiadas pela Associação Médica Brasileira 56 ^,^ 57 . 

 Estudo desenvolvido entre 1995 e 1999 no Hospital Naval Marcílio Dias (Rio de Janeiro) analisou 18.690 pacientes utilizando a estratificação de risco de Caprini, sendo 5% deles classificados como de alto risco, 43% como intermediário, e 52% como de baixo risco. Os autores observaram que a profilaxia recomendada foi adotada em 47% dos pacientes de alto risco, que 33% dos pacientes de risco moderado não receberam profilaxia, e que 4,6% dos pacientes de baixo risco receberam profilaxia medicamentosa, embora não houvesse indicação 58 . Os quatro anos desse estudo piloto confirmaram a viabilidade e o valor do registro e mostraram um aumento considerável no uso de HBPM, associado à redução de seis vezes na incidência de TEV sintomático. 

 Um novo registro multicêntrico foi realizado para investigar a incidência e a distribuição de fatores de risco para TEV em pacientes clínicos, cirúrgicos e obstétrico-ginecológicos internados e o uso de profilaxia no Brasil 59 . No período entre 1999 e 2001, foram coletados dados de 27.450 pacientes. Os registros mostraram que aproximadamente um quarto dos pacientes de alto risco e metade dos de moderado risco não receberam tromboprofilaxia, provavelmente devido à falta de conhecimento dos fatores de risco e estratégias profiláticas apropriadas. Em dois terços dos pacientes de baixo risco não foi realizada qualquer medida profilática. 

 Pereira et al. realizaram estudo prospectivo com 850 pacientes internados em um Hospital de Roraima para constatar se a profilaxia para TVP era utilizada de forma adequada, segundo o modelo de Caprini 60 . Do total, 67% eram pacientes clínicos e 58% considerados de médio e alto risco para desenvolver TVP. Dos pacientes que necessitavam tromboprofilaxia, apenas 24% a receberam e apenas em 20% foi considerada adequada. Os autores concluíram que eram necessários programas de educação continuada. 

 Rocha et al. 61 ^,^ 62 tinham como objetivo implementar um programa de profilaxia de TEV voltado para pacientes clínicos, com a criação de uma comissão, realização de palestras e distribuição de algoritmos baseados na diretriz brasileira e avaliar seu impacto na adequação da tromboprofilaxia em hospitais de Salvador em três etapas. Na primeira etapa, em 2005, foi avaliada a utilização e adequação da profilaxia nos pacientes clínicos internados. Na segunda etapa, a partir de 2007 e por 12 meses, foi implantado um programa de profilaxia. E na última etapa, em 2008, o impacto do programa foi avaliado. Os autores concluíram que os fatores de risco são frequentes em pacientes clínicos, que existe ampla variação na profilaxia prescrita em hospitais públicos e privados, e que apenas a minoria dos pacientes clínicos hospitalizados e candidatos à profilaxia recebem dosagem adequada. Concluíram também que aulas de educação continuada e distribuição passiva de algoritmos de profilaxia para TEV são insuficientes para melhorar a utilização da profilaxia, mas melhoram a sua adequação 61 ^,^ 62 . 

 Okuhara et al. 63 desenvolveram um estudo com 296 pacientes internados submetidos a procedimentos cirúrgicos vasculares e ortopédicos, para determinar a incidência de TVP e a qualidade de profilaxia utilizada. A incidência global de TVP foi de 7,5%. Quanto aos grupos de risco, 15% foram considerados de risco moderado, 24% de alto risco e 50% de altíssimo risco. A profilaxia foi correta em apenas 58%. As taxas de profilaxia adequada foram de 72% tanto no grupo de alto como no de altíssimo risco. O uso excessivo de farmacoprofilaxia foi evidenciado em 69% e 61% nos grupos de baixo e moderado risco, respectivamente. Embora a maior parte dos pacientes seja considerada de alto e altíssimo risco, na prática médica essa aplicação continua deficiente 63 . 

 Por fim, o mais recente estudo transversal com análise de prontuários realizado em Curitiba e publicado em 2017 comparou o uso ou não da profilaxia nas especialidades clínicas e cirúrgicas de acordo com seu fator de risco para TEV e mostrou que apenas 66% dos pacientes receberam profilaxia. Dentre os pacientes clínicos, 93% receberam contra 44% do grupo cirúrgico. Pacientes clínicos de moderado a alto risco receberam mais profilaxia que os cirúrgicos 64 . 

##  ESTRATÉGIAS PARA AUMENTAR A ADERÊNCIA ÀS DIRETRIZES E AO USO APROPRIADO DA TROMBOPROFILAXIA 

 Vários estudos evidenciam que o alerta aos profissionais da saúde sobre os pacientes com risco de TEV aumenta a probabilidade do uso de profilaxia. 

 Muitas abordagens e estratégias existem. Educação didática simples ou disseminação passiva de diretrizes baseadas apenas em evidências não são efetivas 65 ^,^ 66 . São necessárias múltiplas e repetitivas técnicas de conteúdo educacional, sistema de alertas como lembretes de avaliação de risco e auditorias, sendo a combinação desses sistemas parece ser, de fato, mais efetiva. Uma das maneiras para aumentar a eficácia e o uso de tromboprofilaxia é a implantação de sistemas de alertas eletrônicos, já usados há 17 anos. Um ensaio controlado randomizado, publicado em 2001, em que um sistema computadorizado lembrava automaticamente os médicos da necessidade da profilaxia em pacientes hospitalizados, mostrou que a heparina profilática era administrada em 32,2% dos pacientes no grupo dos profissionais que utilizavam o sistema eletrônico e em 18,9% dos pacientes no grupo que não o usavam 67 . 

 No Reino Unido, o *Clinical Decision Support* (CDS), um sistema baseado em computador, tem sido uma forma de solicitar e orientar profissionais para prescrever profilaxia apropriadamente. Modificações na adesão deles com lembretes nos computadores ainda são modestas 68 , mas alertas eletrônicos e CDS computadorizados têm aumentado a prescrição em pacientes clínicos hospitalizados 69 ^,^ 70 . 

 Estudo transversal em duas fases (antes e após implantação de um novo protocolo de profilaxia para TEV) foi realizado em hospital de Porto Alegre para avaliar o impacto da implantação na profilaxia de um sistema informatizado de suporte à decisão clínica combinado a seminários de orientação. A adequação da profilaxia aumentou de 46,2% para 57,9% quando foram comparados os dois períodos, principalmente em pacientes com câncer (de 18,1% para 44,1%) e naqueles com três ou mais fatores de risco (de 25% para 42,9%) 71 . 

 Apesar de muitas tentativas e do aumento nas avaliações de risco de TEV, a profilaxia é subutilizada até hoje, e existem algumas evidências da pequena aderência às diretrizes publicadas 72 ^,^ 73 . 

 Lau & Haut conduziram uma pesquisa MEDLINE para identificar estudos publicados entre 2001 e 2012 que avaliavam os diversos tipos de intervenções para melhorar o uso de profilaxia de TEV em pacientes hospitalizados, de acordo com os seguintes parâmetros: educação somente, baseada em papéis, computadorizada, auditoria em tempo real, ou combinação de intervenções 74 . 

 Fortes evidências de vários estudos de alta qualidade demonstram a efetividade da profilaxia do TEV para populações especificas; entretanto, é preciso estratificação de risco para assegurar que a profilaxia seja direcionada a pacientes apropriados e, mesmo assim, as taxas de profilaxia permanecem sub-ótimas e o TEV continua a ser um problema na segurança dos pacientes. Além disso, existem poucas evidências de quais intervenções específicas são efetivas para aumentar essas taxas de profilaxia. Educação apenas dos profissionais não é o melhor mecanismo para aumentar o uso da profilaxia. 

 Evidências, embora baseadas em estudos não randomizados sem pacientes controles e, portanto, considerados de baixa qualidade, apoiam que educação combinada com outras estratégias de melhoria de qualidade e abordagens tecnológicas, como alertas e apoio à decisão clínica informatizada obrigatória, provavelmente promova mais a prática do uso da profilaxia, evitando danos aos pacientes com TEV.
